# The effect of endoscopic submucosal dissection in patients with early gastrointestinal cancer and precancerous lesion

**DOI:** 10.12669/pjms.41.5.11678

**Published:** 2025-05

**Authors:** Nan Cao, Yang Zhao, Yanna Ma, Chunxiao Cao, Haiguo Zhang

**Affiliations:** 1 Nan Cao Department of Oncology, Zunhua People’s Hospital, Zunhua, Hengyang, Hebei Province 064200, P.R. China; 2 Yang Zhao Department of Gastroenterology, Zunhua People’s Hospital, Zunhua, Hengyang, Hebei Province 064200, P.R. China; 3 Yanna Ma Emergency Department Catheter Maintenance Clinic, Zunhua People’s Hospital, Zunhua, Hengyang, Hebei Province 064200, P.R. China; 4 Chunxiao Cao Department of Oncology, Zunhua People’s Hospital, Zunhua, Hengyang, Hebei Province 064200, P.R. China; 5 Haiguo Zhang Department of Critical Care Medicine, Zunhua People’s Hospital, Zunhua, Hengyang, Hebei Province 064200, P.R. China

**Keywords:** Endoscopic submucosal dissection, Gastrointestinal cancer, Precancerous lesion

## Abstract

**Objective:**

To explore the efficacy and safety of endoscopic submucosal dissection (ESD) in patients with early gastrointestinal (GI) cancer and precancerous lesion.

**Methods:**

A total of 132 patients with early GI cancer or precancerous lesion who underwent surgery at Zunhua People’s Hospital between March 2021 and June 2023 were retrospectively analyzed. Among them, 65 patients underwent endoscopic mucosal resection (EMR) (Control group), and 67 underwent ESD (ESD group). Perioperative information, treatment outcomes, stress response indicators, miR-146a and miR-199a levels, and incidence of postoperative complications were compared between the two groups.

**Results:**

Surgery duration in the ESD group was significantly longer than that in the Control group. However, the intraoperative blood loss in the ESD group was lower, and the duration of anal ventilation and hospitalization was longer than in the Control group (*P*<0.05). Total treatment efficacy of the ESD group was significantly higher compared to the Control group (*P*<0.05). After the surgery, levels of serum cortisol (Cor), norepinephrine (NE), epinephrine (E), and aldosterone (ALD) in both groups significantly increased compared to those before the surgery, and were significantly lower in the ESD group compared to the Control group (*P*<0.05). After the surgery, expression levels of miR-146a in both groups significantly decreased, while miR-199a levels significantly increased compared to the preoperative levels, and the difference in the ESD group was more significant than in the Control group (*P*<0.05). There was no statistically significant difference in the incidence of complications between the two groups (*P*>0.05).

**Conclusions:**

In patients with early GI cancer or precancerous lesion, ESD can reduce surgical trauma, improve the expression of miR-146a and miR-199a, and alleviate the degree of surgical stress response. ESD is safe and is associated with improved treatment effectiveness and reduce occurrence of complications.

## INTRODUCTION

Precancerous conditions such as intraepithelial neoplasia and intestinal metaplasia, have a high tendency for malignancy.^1^,^2^ In recent years, with the continuous development of endoscopic technology, early detection rate of cancerous lesion in the gastrointestinal (GI) tract has greatly improved,^3^ positively impacting treatment efficiency and improving the outcomes.^1^–^3^

Surgery is an important treatment option for cancerous lesion of the digestive tract.^4^ However, traditional open surgery is associated with greater trauma and a higher risk of postoperative complications, such as adhesion obstruction, anastomotic stenosis, and infection, which have a negative impact on postoperative quality of life of patients.^4^,^5^ Recently, minimally invasive procedures, such as endoscopic assisted surgery, have become increasingly popular, as they have significant advantages in lowering surgical trauma, reducing the risk of complications, and shortening the postoperative rehabilitation process.^6^,^7^

Endoscopic mucosal resection (EMR) is an endoscopic technique used for the staging and treatment of superficial neoplasms of the GI tract. Endoscopic submucosal dissection (ESD) is a minimally invasive treatment measure that evolved from endoscopic mucosal resection. ESD can completely peel off larger area of diseased tissue, which increases the accuracy of pathological diagnosis and ultimately improves treatment effectiveness and safety.^8^,^9^ However, there are only a few studies comparing outcomes between EMR vs ESD for GI tract. This study aimed to investigate the value of ESD for the treatment of cancerous lesion of the digestive tract.

## METHODS

A total of 132 patients with precancerous lesion of the digestive tract who underwent surgery at Zunhua People’s Hospital between March 2021 and June 2023 were retrospectively included. All procedures performed in the study involving human participants were in accordance with the ethical standards of the institutional and/or national research committee(s) and the Helsinki Declaration (as revised in 2013).

### Ethical Approval:

The study was approved by the Ethics Committee of Zunhua People’s Hospital (Approval No. 202111-397, approved on October 23, 2023). The requirement for informed consent was waived by the ethics committee due to the observational and retrospective nature of the study.

### Inclusion criteria:

- Diagnosis made by digestive endoscopy and pathological biopsy.
- Endoscopy and ultrasonography showing lesions that are limited to the mucosal layer/mild infiltration into the submucosa but do not involve the muscular layer, and met the criteria of early GI or precancerous protuberant lesions as follows:
- Esophageal precancerous lesions: intraepithelial neoplasia, Barrett’s esophagus and squamous cell papilloma;
- Gastric precancerous lesions: intraepithelial neoplasia, intestinal metaplasia;
- Precancerous lesions of colorectal cancer: adenoma, adenomatosis and dysplasia associated with inflammatory bowel disease;
- Early esophageal cancer: lesions were limited to the mucosa or submucosal layer, without invading the muscularis layer and without lymph node metastasis;
- Early gastric cancer: lesions were limited to the mucosa and submucosa regardless of tumor area and lymph node metastasis;
- Early colorectal cancer: intramucosal and submucosal cancer, without lymph node metastasis.
- Lesions are located in the gastric body.
- 18 years ≤ age ≤ 70 years.
- Complete clinical data.

### Exclusion criteria:

- Other diseases of digestive system.
- Patients with diseases of blood system and immune system.
- Patients with other malignant tumors.
- Women during lactation and pregnancy.
- Organic lesions in important organs.
- Lymph node metastasis and distant metastasis occurred in tumor lesions.

### Preoperative preparation:

Fasting and water deprivation were initiated six hours before the operation. Ethylene glycol was used to clean the intestine.

### EMR:

Ultrasound examination was performed to accurately locate the lesions, and to assess its volume. Skin and subcutaneous tissue were cut, pathological changes were evaluated, total or subtotal resection, subtotal resection, and partial resection were performed, and vital signs were closely monitored during the surgery. In the case of any abnormality, the doctor was immediately informed and corresponding treatment measures were taken. The abdominal cavity was closed after no active bleeding occurred in the operation area, and the subcutaneous tissue and skin were sutured step-by-step. Antibiotics were routinely administered postoperatively.

### ESD:

Criteria used to assess lesions for ESD eligibility were as follows:

### Esophageal lesions:

Lesion infiltration was limited to the M1 and M2 layers, diameter <25 mm, no lymphatic invasion;

### Gastric lesions:

differentiated intramucosal carcinoma without ulcer; differentiated intramucosal carcinoma with ulcer and diameter ≤ 30mm; differentiated carcinoma with diameter ≤ 30mm without ulcer and lymph node metastasis; poorly differentiated intramucosal carcinoma with diameter < 20mm;

Suspected early invasive colorectal cancer; colorectal intraepithelial lesions with a lesion diameter >20 mm; associated fibrotic lesions, such as adenomas occasionally seen in inflammatory bowel disease; recurrent or residual lesion tissue after internal diameter resection (tissue internal diameter >10 mm).

The lesions were located by endoscopy. Argon ion electrocoagulation was performed at about three mm around the lesion for electrocoagulation labeling. Mixed solution of indigo carmine 3ml+hyaluronic acid 5ml+normal saline 95 ml was injected at multiple points under the lesion mucosa along the outer edge of the marker until the lesion was effectively bulged. A needle knife was used to cut the lateral mucosa along the outer edge of the mark. The base of the lesion was stripped after mucosal incision, and the mixture was continuously injected under the mucosa to ensure the uplifting of the lesion. After lesion dissection, electrocoagulation was performed using a hemostatic clip or argon ion coagulation. Care was taken to ensure that there is no exudate in the wound. Gas was cleared from the cavity, and the endoscope was retracted. Antibiotics were routinely administered after the operation.

### Observation indexes included:

- Perioperative conditions, such as operation duration, intraoperative blood loss, anal exhaust time, and length of hospital stay.
- Treatment effectiveness. Classification was as follows: Clinical cure - clinical symptoms disappeared, and no new lesions were detected three months after the operation; Effective- clinical symptoms were relieved but did not disappear, and no new lesions were detected three months after the operation; Ineffective- no clinical symptoms were relieved, and cancerous tissue spread was detected around the resection site three months after the operation. Effective and Clinical cure were included in the total effective rate.
- Stress response index. Levels of Cor, NE, E, and ALD were measured in venous blood. Cor and ALD were determined by radioimmunoassay; NE and E were determined by electrochemiluminescence.
- MiR-146a, miR-199a levels were determined by quantitative reverse transcription polymerase chain reaction (RT-qPCR). Briefly, serum cells were washed with PBS three times and RNA was extracted using Trizol (Manufacturer: Beyotime; China)). RT-PCR was performed using 48/96T kit. RT-qPCR was performed using GoTaq^®^ qPCR Master Mix and a RT-qPCR PCR System. The reaction conditions were as follows: denaturation at 95 °C for 10 minutes, followed by 40 cycles of 95 °C, 58 °C, and 72 °C for 10 minutes, 20 seconds, and 10 seconds, respectively. Quantitative PCR reaction was performed on miR-146a and miR-199a. The primers sequence for miR-146a was: upstream: 5 ′ - CGTGCGACGTGAGTGCA-3 ′, downstream: 5 ′ - TTAGTGAGCGCGGATGA-3 ′; MiR-199a Primer Sequence: Upstream: 5 ′ - GAATTCTGGAACTACCCACAAACC-3 ′, Downstream: 5 ′ - GCGGCGCTAGCAGGAGGAAGTAAGG-3 ′, miR-146a and miR-199a expression were calculated (Manufacturer: Shanghai Kexing Biotechnology Co., Ltd.; China). 5) Incidence of complications including perforation, infection, bleeding, and gastric stenosis.

### Statistical Analysis:

All data were analyzed by spss25.0 software (IBM Corp, Armonk, NY, USA). The normality of the data was evaluated using the Shapiro-Wilk test. Normally distributed data were expressed as the mean ± standard deviation. An independent sample t-test was used for intergroup comparisons, and a paired t-test was used for intragroup comparisons. The counting data were expressed as the number of cases and compared using Chi-square test. P<0.05 indicated statistically significant difference.

## RESULTS

A total of 132 patients (73 males and 59 females) met the inclusion criteria. Among them, 67 patients underwent ESD, and 65 underwent EMR. There was no significant difference in the baseline data between the two groups (*P*>0.05) (Table-I). Operation time in the ESD group was significantly longer than that in the control group, while the intraoperative blood loss was lower, and the anal exhaust time and hospitalization time of the ESD group were shorter than those of the control group (*P*<0.05) (Table-II). The total effective rate in the ESD group was significantly higher than that in the control group (*P*<0.05) (Table-III). Before the surgery, there was no significant difference in serum stress response indices between the two groups (*P*>0.05).

**Table-I:** Comparison of baseline data between the two groups.

Baseline data	Control group (n=65)	ESD group(n=67)	χ^2^/t	P
Gender (male/female)	38/27	35/32	0.517	0.472
Age (years)	53.20±5.96	52.10±6.34	1.023	0.308
Lesion diameter (cm)	2.82±0.48	2.74±0.53	0.888	0.376
BMI (kg/m²)	23.46±2.87	22.58±2.97	1.740	0.084
Diabetes mellitus (yes)	11 (16.92)	14 (20.90)	0.339	0.560
Hypertension (yes)	16 (24.62)	20 (29.85)	0.456	0.500
Smoking (yes)	36 (55.38)	32 (47.76)	0.768	0.381
drink (yes)	35 (53.85)	30 (44.78)	1.086	0.297

**Table-II:** Comparison of perioperative conditions between the two groups.

Group	Operation time (minute)	Intraoperative blood loss (ml)	Anal exhaust time (hours)	hospitalization time (days)
Control group (n=65)	41.03±5.72	126.08±14.45	20.15±3.43	8.91±2.35
ESD group(n=67)	60.03±8.54	105.25±13.61	17.55±2.94	7.64±2.05
*t*	-15.058	8.526	4.684	3.301
*P*	<0.001	<0.001	<0.001	0.001

**Table-III:** Comparison of therapeutic effects between the two groups.

Group	Clinical cure	Effective	Ineffective	Total effective rate
Control group (n=65)	30 (46.15)	23 (35.38)	12 (18.47)	53 (81.54)
ESD group(n=67)	39 (58.21)	25 (37.31)	3 (4.48)	64 (95.52)
*χ^2^*				6.628
*P*				0.036

After the operation, serum levels of COR NE, E, and ALD in the two groups were significantly higher than those before surgery, but were markedly lower in the ESD group compared to the control group (*P*<0.05) (Table-IV). There was no significant difference in the expression of miR-146a and miR-199a between the two groups before the operation (*P*>0.05). After the surgery, expression levels of miR-146a and miR-199a were significantly different between the two groups before operation (P>0.05). Postoperative expression levels of miR-146a and miR-199a in the two groups were lower than before the operation, and significantly lower in the ESD group compared to the control group (*P*<0.05) (Table-V). There was no statistically significant difference in the incidence of complications between the two groups (*P*>0.05) (Table-VI).

**Table-IV:** Comparison of stress response indexes between the two groups.

Time	Group	Cor (ug/L)	NE (mmol/L)	E (mmol/L)	ALD (pg/ml)
Before operation	Control group (n=65)	179.26±20.40	0.69±0.22	0.51±0.18	30.77±7.10
	ESD group (n=67)	181.76±23.17	0.72±0.22	0.53±0.16	31.96±6.31
	*t*	-0.658	-0.824	-0.616	-1.017
	*P*	0.512	0.411	0.539	0.311
After operation	Control group (n=65)	232.32±21.28^a^	1.32±0.24^a^	0.87±0.20^a^	45.79±7.12^a^
	ESD group (n=67)	218.40±18.80^a^	1.19±0.19^a^	0.72±0.15^a^	40.74±5.55^a^
	*t*	3.983	3.610	4.787	4.551
	*P*	<0.001	<0.001	<0.001	<0.001

*Note:* Compared with that before operation in the same group,

a *P*<0.05.

**Table-V:** Comparison of miR-146a and miR-199a between the two groups

Time	Group	miR-146a	miR-199a
Before operation	Control group (n=65)	1.82±0.19	1.66±0.30
	ESD group(n=67)	1.79±0.20	1.71±0.31
	*t*	0.972	-0.909
	*P*	0.333	0.365
After operation	Control group (n=65)	1.24±0.18^a^	1.17±0.27^a^
	ESD group(n=67)	1.02±0.15^a^	1.04±0.25^a^
	*t*	7.699	2.671
	*P*	<0.001	0.009

*Note:* Compared with that before operation in the same group,

a *P*<0.05.

**Table-VI:** Comparison of complications between the two groups.

Group	Perforation	Infection	Bleeding	Gastric stenosis	Total incidence
Control group (n=65)	3 (4.62)	2 (3.08)	2 (3.08)	1 (1.54)	8 (12.31)
ESD group(n=67)	0 (0.00)	0 (0.00)	1 (1.49)	1 (1.49)	2 (2.99)
*χ^2^*					2.872
*P*					0.090

## DISCUSSION

The results of this study showed that ESD is associated with significantly reduced surgical trauma and quicker functional rehabilitation in patients with GI lesions compared to EMR. Our results suggest that ESD is able to improve the treatment effect and promotes the recovery. Nishizawa T et al.^10^ confirmed that ESD can effectively remove lesions, maintain gastric function, and improve the quality of life of patients with early gastric cancer. However, the study also pointed out that patients with early gastric cancer still had a risk of recurrence after ESD treatment.^10^ Esaki et al.^11^ used ESD to treat patients with early gastric cancer, and showed that it can shorten the time of related treatments, which is imperative for improving the effectiveness and safety of therapy. Pagano et al.^12^ evaluated the application value of ESD in the treatment of precancerous and malignant epithelial tumors of the digestive tract, and demonstrated a success rate of 99.1%, and the incidence of complications of only 9.3% (including 5.6% massive hemorrhage and 3.7% perforation). In addition, Chen et al.^13^ found that the complete resection and subtotal resection rates of ESD in gastrointestinal neuroendocrine tumors can reach 100%, while the incidence of perforation and bleeding can be as low as 1.3% and 2.7%, respectively.

Moreover, during the treatment, there were no cases of distant or lymph node metastases. This research is consistent with the results and conclusions of our study.^10^–^13^ We may suggest that ESD can be preferred over open surgery for patients with ESD indications due to its superior treatment effect and faster rehabilitation of body function.^14^,^15^ We may speculate that the observed high efficiency of ESD may be due to the fact that this method allows to develop a personalized treatment scheme based on the lesion volume and specific location. Even large lesions, or lesions with scars and other irregularities can be completely stripped from the surface of the muscularis propria at one time.^15^,^16^ At the same time, compared with EMR, ESD is associated with less trauma and is a simple operation that allows accurate and fast removal of the lesion, and improves complete and curative resection rates. Additionally, ESD will ultimately minimize trauma caused by invasive operations and its subsequent impact on the postoperative rehabilitation process.^2^,^17^

Nevertheless, ESD is still an invasive treatment, which can lead to different degrees of stress reactions in patients.^18^ In this study, serum levels of COR, NE, E, and ALD in patients who underwent ESD were lower than those in the control group. Our results show that compared with EMR, ESD can reduce the degree of stress reactions in patients with cancerous lesion of the digestive tract during the surgery. We may speculate that during ESD, injection of mixed solutions can uplift the lesion site, effectively separating the submucosal muscle tissue. That allows to accurately and completely peel off the lesion tissue, minimizing injury and stress reactions.^18^,^19^ Draganov PV et al.^20^ also showed that ESD allows to accurately peel large lesions, and the depth of resection can reach the muscularis propria.

It results in complete resection of lesions with large areas and deep invasion, and can provide complete pathological data and guidance for follow-up intervention programs. In addition, submucosal dissection can provide a clear surgical field, facilitate bypassing of small blood vessels, and reduce perforation and bleeding while completely removing the lesion. Furthermore, electrocoagulation can maintain the blood supply around the lesion and reduce deep mucosal injury.^20^ Our results demonstrated differences in the expression of miR-146a and miR-199a miRNAs between normal and tumor tissues. Moreover, miRNA expression in tissues was stable and specific, and therefore, may be used for prognosis evaluation.^21^,^22^ MiR-146a plays a regulatory role in the occurrence and progression of tumor diseases.^23^

It can mediate cell-directed metastasis and chemotaxis and activate expression of chemokines in epithelial cells, neutrophils, endothelial cells, and lymphocytes. Abnormal expression of miR-146a was shown to enhance the ability of tumor cells to adhere to endothelial cells and promote disease progression.^23^ MiR-199a plays a two-way regulatory role in the pathophysiological processes of gastrointestinal tumors. A decrease in its expression can promote tumor invasion and progression and activate epithelial mesenchymal transition.^24^ The results of this study showed that miR-146a and miR-199a levels in the postoperative ESD group were lower than those in the control group. These results suggest that ESD is also beneficial in regulating the expression of miR-146a and miR-199a, which further ensures a good prognosis of the disease.

### Limitations:

First, this was a single-center retrospective study. Second, the sample size and observation indexes were limited. Third, there was no postoperative follow-up. Further studies with a longer follow-up time are needed to verify the results. Finally, the effects of the two methods on the long-term functional recovery of the patients were not analyzed. Higher-quality research is required to verify our conclusion.

## CONCLUSION

Compared to EMR, ESD can reduce surgical trauma, improve the expression of miR-146a and miR-199a, and reduce the degree of surgical stress response in patients with early GI cancer and precancerous lesion, which can improve the therapeutic effect and ensure safety.

### Authors’ Contributions:

**NC:** Study design, literature search manuscript writing, revision and validation and is responsible for the integrity of the study.

**YZ, YM, CC and HZ**: Data collection, data analysis and interpretation. Critical Review.

All authors have read, approved the final manuscript and are accountable for the integrity of the study.
